# Preterm birth is associated with xenobiotics and predicted by the vaginal metabolome

**DOI:** 10.1038/s41564-022-01293-8

**Published:** 2023-01-12

**Authors:** William F. Kindschuh, Federico Baldini, Martin C. Liu, Jingqiu Liao, Yoli Meydan, Harry H. Lee, Almut Heinken, Ines Thiele, Christoph A. Thaiss, Maayan Levy, Tal Korem

**Affiliations:** 1 Program for Mathematical Genomics, Department of Systems Biology, Columbia University Irving Medical Center, New York, NY USA; 2 Department of Biomedical Informatics, Columbia University Irving Medical Center, New York, NY USA; 3 School of Medicine, University of Ireland, Galway, Galway, Ireland; 4 Discipline of Microbiology, University of Galway, Galway, Ireland; 5 Ryan Institute, University of Galway, Galway, Ireland; 6 APC Microbiome Ireland, University College Cork, Cork, Ireland; 7 Department of Microbiology, Perelman School of Medicine, University of Pennsylvania, Philadelphia, PA USA; 8 Institute for Diabetes, Obesity, and Metabolism, Perelman School of Medicine, University of Pennsylvania, Philadelphia, PA USA; 9 Institute for Immunology, Perelman School of Medicine, University of Pennsylvania, Philadelphia, PA USA; 10 Department of Obstetrics and Gynecology, Columbia University Irving Medical Center, New York, NY USA; 11 CIFAR Azrieli Global Scholars program, CIFAR, Toronto, Ontario Canada

**Keywords:** Microbiome, Metabolomics, Machine learning, Diseases, Risk factors

## Abstract

Spontaneous preterm birth (sPTB) is a leading cause of maternal and neonatal morbidity and mortality, yet its prevention and early risk stratification are limited. Previous investigations have suggested that vaginal microbes and metabolites may be implicated in sPTB. Here we performed untargeted metabolomics on 232 second-trimester vaginal samples, 80 from pregnancies ending preterm. We find multiple associations between vaginal metabolites and subsequent preterm birth, and propose that several of these metabolites, including diethanolamine and ethyl glucoside, are exogenous. We observe associations between the metabolome and microbiome profiles previously obtained using 16S ribosomal RNA amplicon sequencing, including correlations between bacteria considered suboptimal, such as *Gardnerella* *vaginalis*, and metabolites enriched in term pregnancies, such as tyramine. We investigate these associations using metabolic models. We use machine learning models to predict sPTB risk from metabolite levels, weeks to months before birth, with good accuracy (area under receiver operating characteristic curve of 0.78). These models, which we validate using two external cohorts, are more accurate than microbiome-based and maternal covariates-based models (area under receiver operating characteristic curve of 0.55–0.59). Our results demonstrate the potential of vaginal metabolites as early biomarkers of sPTB and highlight exogenous exposures as potential risk factors for prematurity.

## Abstract (web summary)

Characterization of the vaginal microbiome and metabolome reveals that vaginal metabolites, including several exogenous xenobiotics, are predictive of spontaneous preterm birth.

## Main

Preterm birth (PTB), childbirth before 37 weeks of gestation, is the leading cause of neonatal death, and may lead to a variety of lifelong morbidities^1,2^. PTB also reflects a notable racial disparity, manifesting in a substantially higher PTB rate in Black women^3^. This disparity is driven by various factors, such as the persistent stress of systemic and environmental racism and a lack of access to maternal care^4^. Spontaneous preterm birth (sPTB), PTB not medically induced, accounts for two-thirds of all PTBs^1^. Despite extensive efforts, methods for early prediction, prevention or treatment of PTB are lacking^1,5,6^, and its prevalence remains high^1^.

The human microbiome is a strong biomarker of many complex diseases^7–11^. The vaginal microbiome, specifically, has been repeatedly associated with sPTB and other adverse pregnancy outcomes^12–17^. However, a clear consensus on the relationship between the vaginal microbiome and sPTB has yet to emerge^18^, and our knowledge of specific mechanisms underlying potential host–microbiome interactions in sPTB is lacking.

Metabolites produced or modified by the microbiome have emerged as a prominent factor with potential local and systemic effects on the host^19–22^. Their study has been facilitated by metabolomics, which enables the measurement of thousands of small molecules present in an ecosystem, and paired microbiome–metabolome studies have yielded potential mechanistic insights into host–microbiome interactions in various pathologies^23,24^. A few studies of the vaginal metabolome described associations with the microbiome, inflammation and PTB^25,26^. However, studies of demographic groups at high risk for sPTB, with measurements of a broad set of metabolites and which generate robust prediction models for sPTB, are still needed to advance our understanding of the role of the vaginal ecosystem in prematurity and other pregnancy outcomes.

Here, we measured the second-trimester vaginal metabolome of 232 pregnant women, for whom the microbiota was previously characterized using 16S ribosomal RNA gene amplicon sequencing^14^. We show that the vaginal metabolome partially corresponds to community state types (CSTs), reveal associations between metabolites measured in the middle of pregnancy and subsequent sPTB, and propose that some of these metabolites are of an exogenous source. Finally, we devise machine learning algorithms that use the vaginal metabolome to predict subsequent sPTB an average of 3 months before delivery, which we validate on two external cohorts. Our results demonstrate a promising approach for studying potential causes of prematurity as well as for early risk stratification, and highlight the need to study environmental exposures as a risk factor for sPTB.

## Results

### Vaginal microbiota and metabolome from a pregnancy cohort

We used mass spectrometry to profile 232 vaginal samples collected between 20 and 24 weeks of gestation from women with singleton pregnancies, for which the microbiota was previously characterized from the same timepoint^14^ (Supplementary Table 1 and Methods). All women with subsequent sPTB and available samples (*N* = 80), as well as similar term birth controls (TB; *N* = 152) were included (Table 1). As expected, PTB history was associated with sPTB (Fisher’s exact *P* = 3 × 10^−4^).

**Table 1:** Cohort characteristics

	sPTB	TB	Difference (*P* value)
***N***	80	152	
**Race (*N*** **(%))**			0.417
**Black**	57 (71.25%)	116 (76.3%)	0.568
**White**	21 (26.25%)	30 (19.7%)	0.331
**Other**	2 (2.5%)	6 (4%)	0.666
**Nulliparous (*N*** **(%))**	29.0 (36.2%)	55.0 (36.2%)	0.894
**PTB history** ***(N*** **(%))**	34 (42.5%)	28 (19.2%)	**0.0003**
**GA at delivery (median weeks (range))**	34 (21–36)	39 (38–39)	**<0.0001**
**BMI (kg** **m**^−^^**2**^ **mean** ± **s.d.)**	30.1 ± 7.8	30.6 ± 7.2	0.65
**Age (years mean** ± **s.d.)**	29 ± 6	28 ± 6	0.28

GA, gestational age; *P*, two-sided Fisher’s exact or Mann–Whitney *U* test. Bold indicates *P* < 0.05.

**Extended Data Fig. 1:**
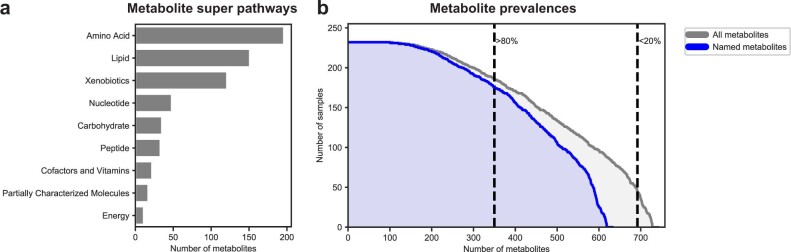
Prevalence and super pathway of assayed metabolites. **a**, Distribution of metabolite super pathways among assayed metabolites. Metabolite super pathway assignments were provided by Metabolon. **b**, Distribution of metabolite prevalences across samples. Gray distribution reflects prevalences of all metabolites (N = 745). Blue distribution only reflects prevalences of named metabolites (N = 635). Dashed lines distinguish metabolites prevalent in more than 80% (N = 352) and more than 20% of samples (N = 694).

**Extended Data Fig. 2:**
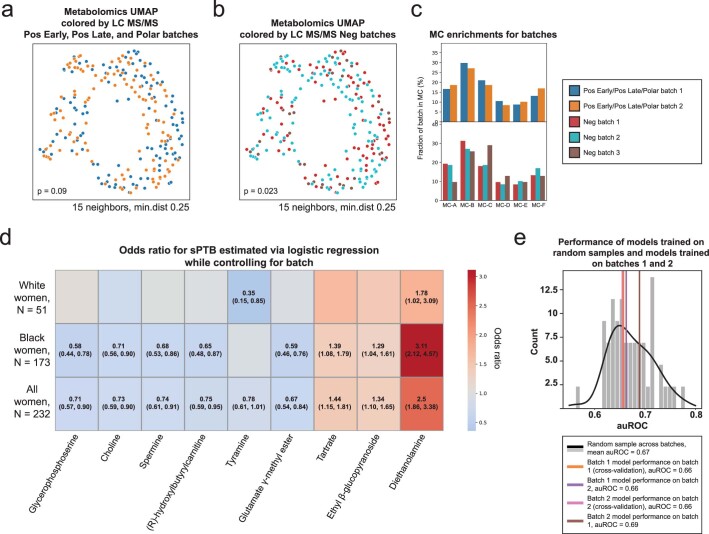
Robustness of analyses to metabolomics batch effects. **a, b**, UMAP ordination of metabolomics data (N = 232), same as Fig. 1b, colored by Pos Early, Pos Late, and Polar platform batches (**a**; 2 batches) and by Neg platform batches (**b**; 3 batches). See Supplementary Table 4 for which metabolites were measured by each platform. Limited batch effect is noted, which is statistically significant only for the 3 batches (PERMANOVA *P* = 0.09 and *P* = 0.023 for 2 and 3 batches, respectively). **c**, The fraction of samples from each batch (y-axis; top, Pos Early, Pos Late, and Polar platform batches; bottom, Neg platform batches) whose metabolite profiles clustered to each metabolite cluster (MC; x-axis), shown for each MC separately. No significant batch effect was detected in MC assignments (Two-sided Fisher’s exact *P* > 0.05 for all without FDR correction). **d**, Heatmap showing odds ratio for sPTB (color bar) for each metabolite from Fig. 2a (x-axis) using a logistic regression model adjusting for batch (according to the appropriate platform for the metabolite, Supplementary Table 4), stratified by maternal race (y-axis). The exact odds ratio and confidence interval are written in the cell for all statistically significant associations (FDR < 0.1). **e**, sPTB classification accuracy (auROC, x-axis) for a prediction model similar to those used for the entire cohort (Fig. 4, Methods), that is: trained and evaluated in cross validation on batch 1 (N = 114; orange; auROC = 0.66; one-sided permutation *P* = 0.44 for lower accuracy than random draw); trained on batch 1 (N = 114) and evaluated on batch 2 (N = 118; violet; auROC = 0.66; *P* = 0.46); trained and evaluated in cross validation on batch 2 (N = 118; magenta; auROC = 0.66; *P* = 0.44); and trained on batch 2 (N = 118) and evaluated on batch 1 (N = 114; brown; auROC = 0.69; *P* = 0.66). Gray histogram (black line, KDE) shows accuracy of models evaluated in cross-validation on random samples (N = 116) from this cohort (mean auROC = 0.67). This analysis demonstrates that a prediction model trained on one of the two batches generalizes well to the other batch, and that both accuracies are to be expected given the limited sample size.

We quantified 635 identified metabolites, as well as 110 unnamed spectral features (Methods). Metabolites belonged to diverse biochemical classes, including amino acids, lipids, nucleotides, carbohydrates and xenobiotics. Most metabolites (549) were measured in over 50% of the cohort, and 108 metabolites were present in all samples (Extended Data Fig. 1; for discussion of batch processing of the samples, see Supplementary Note 1 and Extended Data Fig. 2). We have previously shown that similar measurements are in excellent agreement with measurements by an independent certified medical laboratory^27^.

### The vaginal metabolome partially preserves CST structure

**Fig. 1:**
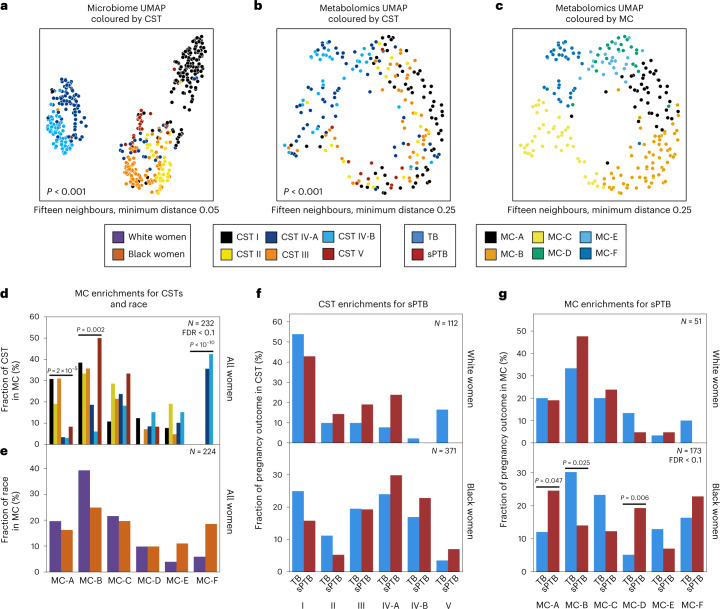
Vaginal metabolome clusters are associated with PTB. **a**–**c**, UMAP ordination of microbiome (**a**, *N* = 503) and metabolomics data (**b** and **c**, *N* = 232), coloured by CSTs (**a** and **b**) or de novo clustering of metabolites data (**c**, MCs; Methods). The vaginal microbiome and metabolome are significantly separated by CSTs (PERMANOVA *P* < 0.001 for both), yet the separation is less clear in the metabolome. For similar plots coloured by maternal race, see also Extended Data Fig. 4c,d. **d**, The fraction of women whose metabolite profiles clustered to each MC, shown for each CST separately. **e**, Similar to **d** but shown for Black and White women separately. **f**, The fraction of White (top) and Black (bottom) women whose microbiomes belonged to each CST, separated by pregnancy outcome. **g**, Similar to **f**, for the fraction of women whose metabolomes clustered to each MC. We show a significant association of sPTB with MCs A, B and D among Black women (*P* = 0.047, *P* = 0.025 and *P* = 0.006, respectively, *q* < 0.1). Number above horizontal lines in **d**–**g** is two-sided Fisher’s exact *P*, *q* < 0.1.

The vaginal microbiome clusters to well-defined CSTs^28^. We demonstrated the same for this cohort^14^ (permutational multivariate analysis of variance (PERMANOVA) *P* < 0.001; Fig. 1a), and investigated whether the vaginal metabolome recapitulates this structure. The metabolome was separated by CSTs (*P* < 0.001; Fig. 1b), and was generally associated with the microbiome (Mantel *P* < 0.001), as previously described^29^. However, specific CSTs were not as well separated. While the metabolomes of women with CST-I (dominated by *Lactobacillus* *crispatus*) and CST-IV (enriched with diverse anaerobes) microbiomes were well separated from the rest of the cohort (PERMANOVA *P* < 0.001 for both), neither the metabolomes of women with CSTs IV-A and IV-B, nor with CST-II (dominated by *Lactobacillus* *gasseri*) and CST-III (dominated by *Lactobacillus* *iners*), were well separated from one another (*P* = 0.158 and *P* = 0.155, respectively). Overall, these results demonstrate a strong but imperfect correspondence between the vaginal microbiome and metabolome.

### Metabolite clusters associate with sPTB

**Extended Data Fig. 3:**
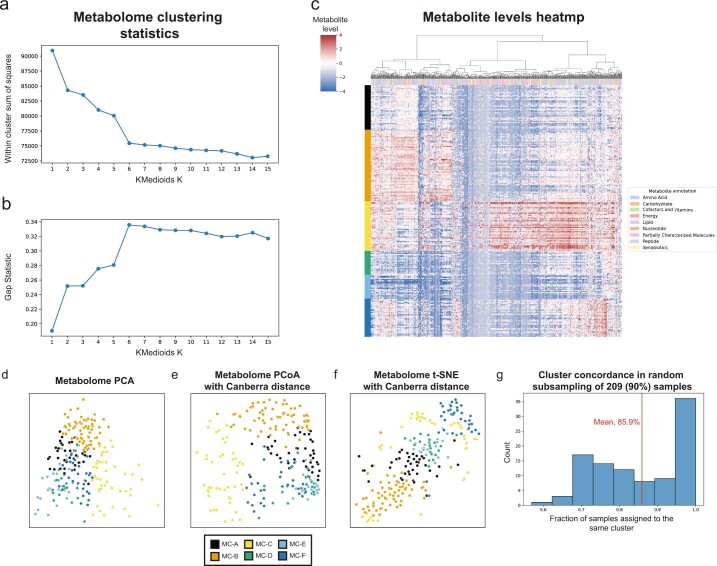
Characteristics of metabolite clusters. **a, b**, Within cluster sum of squared distances (**a**) and gap statistic (**b**) for k-medoids clustering using Canberra distances with k from 1 to 15. A shoulder (**a**) and peak (**b**) are visible for k = 6. **c**, Heatmap showing metabolite levels for each subject (rows) and metabolite (columns). Subjects are sorted by their assigned metabolites cluster (MC) and metabolites are clustered hierarchically using Canberra distance and Ward linkage. The color above each column reflects metabolite annotations (legend to the right). **d-f**, Same as Fig. 1c, using PCA (**d**), Canberra distance-based PCoA (**e**) and t-SNE (**f**). **g**, Histogram of consistency of MC assignment, defined as the fraction of samples assigned to the same MC (x-axis) in 100 iterations in which we randomly selected 90% (209 women) of the cohort, and generated 6 metabolite clusters de novo. The analysis shows that many of the iterations (36 iterations, 36%) had over 95% consistency, with an overall mean consistency of 86%.

**Extended Data Fig. 4:**
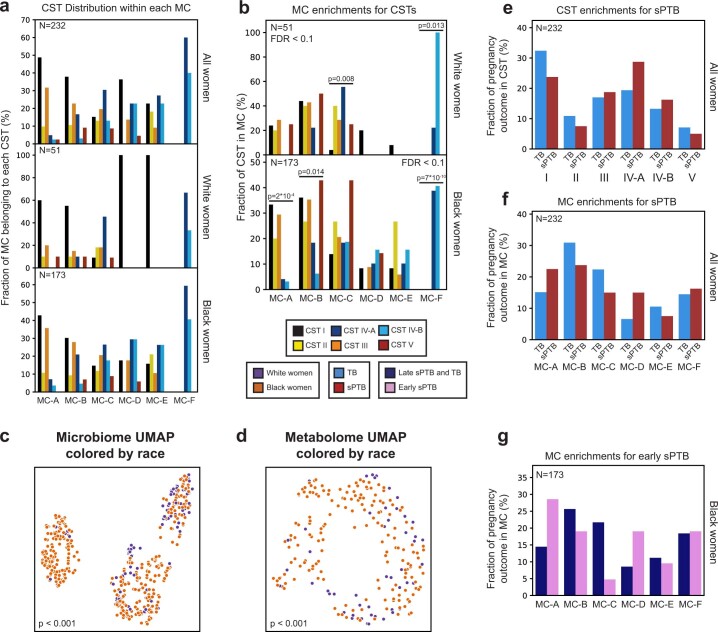
Metabolite clusters correspond to CSTs. **a**, Distribution of CSTs within each metabolite cluster, for all (top; N = 232), White (middle; N = 51) and Black (bottom; N = 173) women. Each group of bars corresponds to a single metabolite cluster and bars within a group sum to 100%. **b**, Same as Fig. 1d, stratified by race. *P -* two-sided Fisher’s exact *p*-values, *q* < 0.1. **c, d**, Same as Fig. 1b, c, colored by maternal race. *P* - PERMANOVA. **e,f**, Same as Fig. 1f, g, performed for all women combined. **g**, Same as Fig. 1g, for association with early sPTB (gestational age at birth < 32).

Next, we performed de novo *k*-medoids clustering of the metabolome, revealing six ‘metabolite clusters’ (MCs A–F; Methods, Fig. 1c, Extended Data Fig. 3 and Supplementary Table 2), which are not as well separated as the separation of the vaginal microbiome to CSTs. The metabolite subpathway most enriched within each MC was polyamine metabolism, dipeptides, dicarboxylated fatty acids, glutamate metabolism, tricarboxylic acid cycle and dipeptides for MCs A–F, respectively (Fisher’s exact *P* < 0.05 for all). Amino-acid-related metabolites were similarly enriched in MCs A,B and D (*P* < 0.01, *q* < 0.1 for all), and xenobiotics in MC-C (Fisher’s exact *P* = 0.005, *q* < 0.1). While MCs A–D are mostly paired with *Lactobacillus*-dominated CSTs (54–93%), MC-F is composed entirely of CST-IV, and MC-E is evenly split (50% CST-IV; Fig. 1d and Extended Data Fig. 4a). Reciprocally, we found various enrichments of CSTs in MCs (Extended Data Fig. 4b).

Similar to the strong association between the global microbiome signature and self-identified race in this cohort (PERMANOVA *P* < 0.001; Extended Data Fig. 4c), we saw a significant difference between the metabolome of Black and White women (*P* < 0.001; Extended Data Fig. 4d). However, we found only mild differences between these subgroups in their assignments to MCs (Fig. 1e). Interestingly, while CSTs are only weakly associated with sPTB and only in White women (Fisher’s exact *P* = 0.047, *q* = 0.21; Fig. 1f and Extended Data Fig. 4e; similar to a previous analysis^14^), we found that several MCs are significantly associated with sPTB in Black women (*P* = 0.047, *P* = 0.025 and *P* = 0.006, respectively, for MCs A, B and D; *q* < 0.1 for all; Fig. 1g and Extended Data Fig. 4f). However, we observed no significant associations with early PTB (<32 weeks; *q* > 0.1 for all, Extended Data Fig. 4g). Taken together, our results demonstrate that the metabolome structure in this cohort better captures associations with prematurity in Black women than the microbiome structure.

### Multiple metabolites associate with sPTB

**Fig. 2:**
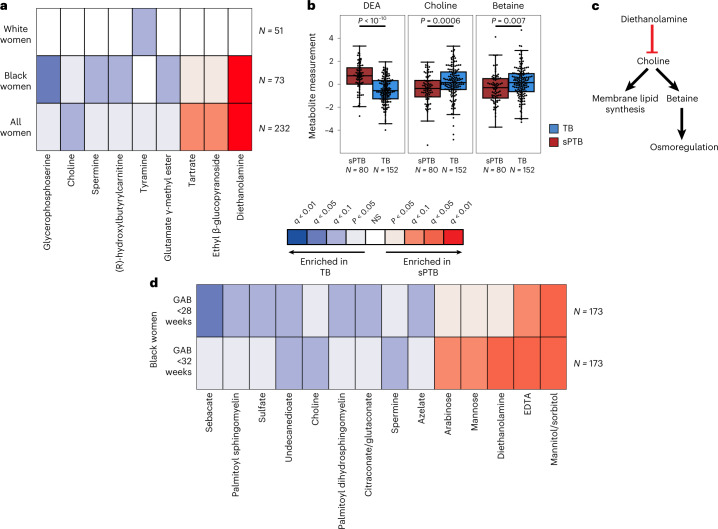
Vaginal metabolites associate with subsequent preterm delivery. **a**, Heat map showing statistically significant associations (two-sided Mann–Whitney *P* < 0.05) between specific metabolite measurements and birth outcomes, stratified by maternal race, and coloured by significance and direction of association. Only metabolites with at least one association with FDR <0.1 are shown. Metabolites are sorted by their average signed (direction of fold change) log *P* value. **b**, Box and swarm plots (line, median; box, IQR; whiskers, 1.5× IQR) of three metabolites with significant associations with sPTB. *P,* two-sided Mann–Whitney *U*. **c**, Illustration summarizing some of the literature regarding the three metabolites shown in **b**. DEA, which is associated with sPTB, was shown to inhibit choline uptake^41^. Choline and betaine, both associated with TB, are important for membrane lipid synthesis and osmoregulation^38,40^. **d**, Same as **a**, with stratification by GAB, performed among Black women. Middle legend applies to **a** and **d**; NS, not significant; *q* < 0.1 indicated by bright colours (legend).

**Extended Data Fig. 5:**
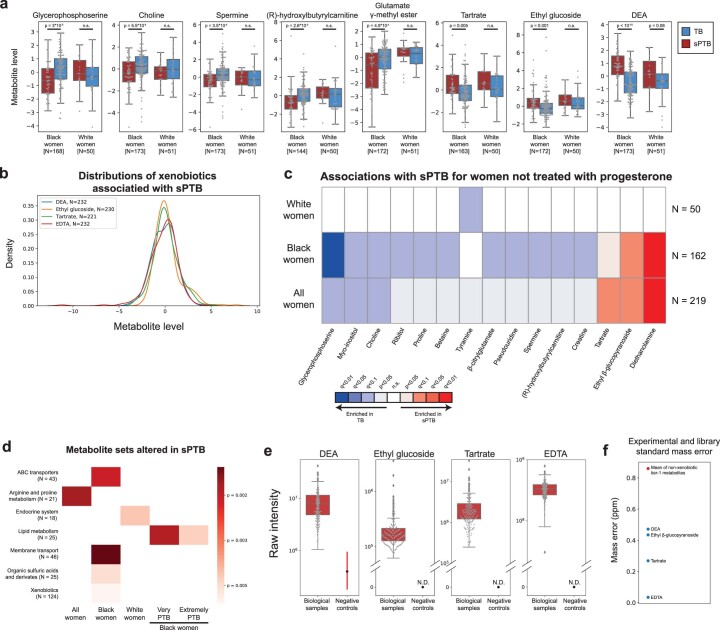
Metabolites altered in sPTB. **a**, Box and swarm plots (line, median; box, IQR; whiskers, 1.5*IQR) of the levels of metabolites associated with sPTB, comparing preterm and term deliveries and stratifying by maternal self-identified race. *P* – two-sided Mann-Whitney *U*. **b**, Distribution (kernel density estimation) of four xenobiotics associated with sPTB or early sPTB across this cohort. Samples with no metabolite detected are excluded. **c**, Same as Fig. 2a, for women not treated with progesterone. **d**, Heatmap showing metabolite sets altered in sPTB in various subsets of this cohort. Colors correspond to two-sided *p*-value of metabolite set enrichment analysis (Methods). Only associations with FDR < 0.1 are shown. **e**, Raw intensity levels measured across samples for the same four xenobiotics as in b, compared to measures from plate negative process controls. Box mid-line, median; box, IQR; whiskers, 1.5*IQR; vertical line, min:max range; dot, mean; N.D., not detected. N = 232 for Diethanolamine; N = 230 for ethyl glucoside; N = 221 for tartrate; N = 232 for EDTA. **f**, Mass error for spectral matching (y-axis) for the same xenobiotics, compared to the mean mass error for all non-xenobiotic, tier 1 metabolites, showing that the four xenobiotic metabolites had very good identification quality.

We next investigated associations between sPTB and specific metabolites. We found four metabolites that are significantly associated with sPTB (Mann–Whitney *P* < 0.05, *q* < 0.1; Fig. 2a and Extended Data Fig. 5a). Three of these, ethyl β-glucopyranoside (ethyl glucoside; *P* = 1.9 × 10^−4^, *q* = 0.065); tartrate (*P* = 4.8 × 10^−4^, *q* = 0.078); and diethanolamine (DEA; *P* < 10^−10^, *q* = 5 × 10^−8^), all higher in sPTB, appear to be of exogenous source^30–36^. We confirmed this using AMON^37^ (Methods), a method that predicts metabolite origins, which predicted that DEA and tartrate were of xenobiotic origin (no prediction could be made for ethyl glucoside; Supplementary Table 3). Of note, DEA is also associated with MC-A (*P* = 0.006, *q* = 0.014) and MC-D (*P* = 0.04, *q* = 0.07), the MCs we found to be enriched with sPTB (Fig. 1g). Despite their likely exogenous source, these metabolites were detected in >95% of this cohort (Extended Data Fig. 5b).

We further found lower levels of choline in women with subsequent sPTB (*P* = 5.5 × 10^−4^, *q* = 0.078; Fig. 2a,b). Choline is an essential nutrient^38^, and lower choline levels were previously found in cord blood from premature infants^39^. Choline is also a precursor of betaine^40^, an osmoregulator that was also negatively associated with sPTB (*P* = 0.007, *q* = 0.29; Fig. 2b). DEA is known to disrupt choline metabolism^41^, and its dermal administration in mice depleted hepatic choline^42,43^. We therefore propose that the higher levels of DEA in sPTB may also be linked to lower choline and betaine levels (Fig. 2b,c). DEA was further shown to be carcinogenic^44^ and teratogenic^42^ in mice. However, the relative nature of our metabolomic assay precludes quantitative comparison with levels measured in previous studies. Taken together, these results highlight a potential role of several metabolites in prematurity, some of which may arise exogenously from environmental exposures.

### Metabolite associations interact with race and sPTB timing

As the metabolome differed between Black and White women, we performed the same association analysis while stratifying by race. Interestingly, we detected five additional metabolites negatively associated with sPTB (Mann–Whitney *P* < 0.05, *q* < 0.1; Fig. 2a and Extended Data Fig. 5a). In Black women, these included glycerophosphoserine (*P* = 3 × 10^−5^, *q* = 0.014), previously reported to be altered in pre-eclampsia^45^; spermine (*P* = 3.5 × 10^−4^, *q* = 0.07), previously shown to be increased in the blood of preterm infants^46^; hydroxybutyl carnitine (*P* = 2.6 × 10^−4^, *q* = 0.065), a ketocarnitine shown to be depleted in the blood of low-birth-weight full-term neonates^47^; and glutamate γ-methyl ester (*P* = 4.9 × 10^−4^, *q* = 0.078). Tyramine, a biogenic amine, was significantly lower in samples from White women who delivered preterm (*P* = 2.8 × 10^−4^, *q* = 0.065; Fig. 2a). Tyramine was shown to co-localize with synaptic vesicles in the mouse uterine plexus, highlighting a possible role in uterine contractions^48^. Altogether, these results highlight the potential connection among vaginal metabolites, metabolite levels in other organs and sPTB.

As several participants in this cohort (*N* = 13, *N* = 11 in Black women) were treated with intravaginal progesterone before or close to sample collection (at weeks 18–23 of gestation), we performed the same analysis only in women not treated with vaginal progesterone. One association, between glutamate γ-methyl ester and TB in Black women (Fig. 2a) no longer passed correction for multiple hypothesis testing (*P* = 0.002, *q* = 0.12; Extended Data Fig. 5c). However, we found an additional seven metabolites to be associated with TB in Black women (all *P* < 0.05; *q* < 0.1; Extended Data Fig. 5c). These included proline (*P* = 6 × 10^−4^, *q* = 0.082), which accounts for about a quarter of the amino acid residues of collagen^49^, and is integral to the extracellular matrix; spermine, a polyamine important for placental angiogenesis^50^, which was lower in Black women with subsequent sPTB (*P* = 4 × 10^−4^, *q* = 0.08) and betaine (*P* = 9 × 10^−4^, *q* = 0.091). *N*-acetylarginine (*P* = 0.0015, *q* = 0.102), which is produced from proline and is necessary for the synthesis of polyamines such as spermine, was also lower in Black women with subsequent sPTB. Both disordered placental angiogenesis and extracellular matrix remodelling have been associated with sPTB^51^.

Earlier preterm deliveries are associated with worse outcomes^1^. Therefore, we next investigated associations between vaginal metabolites and subsequent very and extremely preterm deliveries (gestational age at birth <32 and <28 weeks, respectively). We limited this analysis to Black women, due to their high proportion among such deliveries (21 of 26 and 14 of 15, respectively). We identified 13 metabolites that were associated only with these earlier sPTBs (*P* < 0.05, *q* < 0.1; Fig. 2d). The phospholipids palmitoyl sphingomyelin and palmitoyl dihydro sphingomyelin were both negatively associated with extremely PTB (*P* = 8.7 × 10^−4^, *q* = 0.061 and *P* = 0.0011, *q* = 0.069, respectively). Citraconate was likewise negatively associated with extremely PTB (*P* = 0.0014, *q* = 0.075), and was previously found to have lower concentrations in placental mitochondria of women with severe pre-eclampsia^52^. We also found several sugar or sugar alcohol metabolites to be higher in early PTB, including mannose (*P* = 4 × 10^−4^, *q* = 0.052), previously associated with uropathogens such as *Escherichia* *coli*^53^; arabinose (*P* = 9 × 10^−4^, *q* = 0.061), previously associated with bacterial vaginosis (BV)^54^ and mannitol/sorbitol (*P* = 1.7 × 10^−4^, *q* = 0.022), previously associated with PTB^55^. Ethylenediaminetetraacetic acid (EDTA), an additional xenobiotic whose likely exogenous source^56–58^ was also confirmed by AMON (Methods and Supplementary Table 3), was increased in extremely and very PTB (*P* = 8 × 10^−4^, *q* = 0.061 and *P* = 1.6 × 10^−4^, *q* = 0.044, respectively). EDTA was shown to be cytotoxic in vaginal epithelial cells^59^, and is teratogenic in rats at non-maternotoxic doses^57,60^. EDTA was detected in 100% of women in this cohort (Extended Data Fig. 5b), which is expected given its presence in the sample collection buffer, yet this is unlikely to explain these associations. Overall, we found that metabolite associations with sPTB interact with both race and sPTB timing, and detected an additional sPTB-associated xenobiotic.

### Functional metabolite sets enriched for sPTB associations

We next checked whether functional groups of metabolites (for example, Kyoto Encyclopedia of Genes and Genomes (KEGG) pathways^61^; Supplementary Table 4) are enriched for associations with sPTB, even if changes to any specific metabolite are small (Methods). We found significant enrichment in proline and arginine metabolism (*P* = 0.0018, *q* = 0.058; Extended Data Fig. 5d), consistent with our findings regarding proline and *N*-acetylarginine (Extended Data Fig. 5c). Additionally, and again consistent with the association between tyramine and TB among White women (Fig. 2a), we found an enrichment in metabolites related to the endocrine system among White women (*P* = 0.0045, *q* = 0.077; Extended Data Fig. 5d). We further identified lipid-metabolism-related metabolites to be enriched for associations with early sPTB among Black women (*P* = 0.0019, *q* = 0.032 and *P* = 0.0047, *q* = 0.038 for very and extremely PTB, respectively; Extended Data Fig. 5d), potentially related to other lipid metabolism alterations reported in PTB^62^. Notably, we identified a global enrichment of xenobiotics associated with sPTB among Black women (*P* = 0.006, *q* = 0.054; Extended Data Fig. 5d), consistent with our finding regarding specific metabolites (Fig. 2).

### A network of microbe–metabolite associations in sPTB

**Fig. 3:**
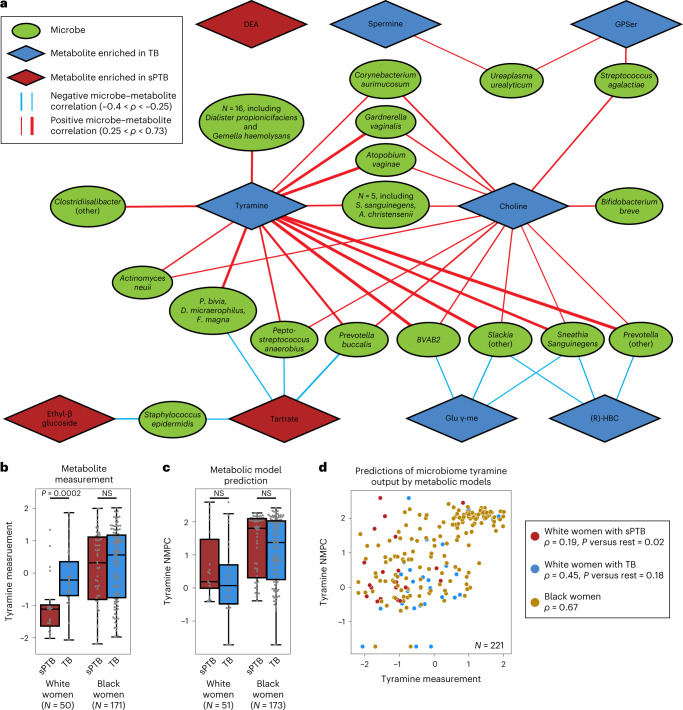
Microbe–metabolite correlations and metabolic models suggest sources for sPTB-associated metabolites. **a**, A network of microbial correlations with metabolites associated with sPTB. Ellipses, microbial species; blue and red diamonds, metabolites enriched in TB and sPTB, respectively; blue and red edges, negative and positive Spearman correlations with FDR < 0.1, |*ρ|* > 0.25, respectively; edge width, median *ρ*. For the same network without grouped nodes, see Extended Data Fig. 6a. **b**,**c**, Box and swarm plots (line, median; box, IQR; whiskers, 1.5× IQR) of tyramine levels, as measured (**b**) and predicted with metabolic models (Methods; **c**), comparing preterm and term deliveries and stratifying by maternal self-identified race. White women who delivered preterm had lower measured vaginal levels of tyramine (*P* = 0.0002), yet our metabolic models predict higher, albeit non-statistically significant, microbiome production of tyramine in women who delivered preterm (*P* = 0.18 and *P* = 0.26 for all and White women, respectively). *P*, Two-sided Mann–Whitney *U*. **d**, Tyramine production derived from microbiome metabolic models (NMPC; Methods; *Y* axis) plotted against measured tyramine levels (*X* axis) and coloured by race and birth outcome (legend). While our models are generally accurate for tyramine (Spearman *ρ* = 0.62, *P* < 10^−10^ across all women), the accuracy for White women who delivered preterm was significantly lower (Spearman *ρ* = 0.19, *P* = 0.02 for comparing correlation strength versus the correlation in other women, two-sided Fisher *R*-to-*z* transform), suggesting a difference in strains, functional capacity, or a non-microbial interaction not captured by our models.

**Extended Data Fig. 6:**
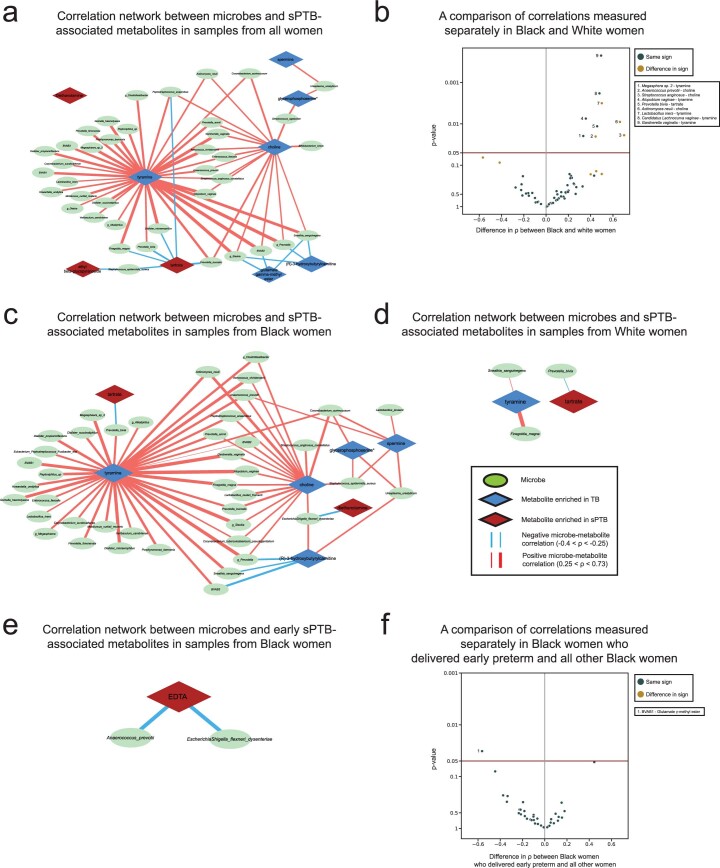
Networks of microbial correlations with PTB-associated metabolites. **a**, Same as Fig. 3a, but with each microbial taxa represented as an individual node. **b**, Volcano plot where every point represents a microbe–metabolite association. X-axis displays the difference between spearman ρ’s calculated separately among Black and White women. Y-axis displays the significance of the difference, using the two-sided Fisher’s R-to-z transform. Horizontal maroon line designates *p* = 0.05. Gold points indicate associations where there is a difference in sign between the correlations among Black and White women. **c, d**, Same as **a**, for associations only among Black (**c**) and White (**d**) women. **e**, Same as **a**, for metabolites associated with extremely or very PTB among Black women. **f**, Same as **b**, for difference in associations between Black women who delivered extremely or very preterm and the rest of the Black women in the cohort.

We next investigated the correlations between the estimated absolute abundances of microbial species and sPTB-associated metabolites (Methods). Contrary to metabolite associations with sPTB, we found weak interactions between microbe–metabolite associations and both race and sPTB timing (Supplementary Note 2). Our results replicate multiple known associations, such as between *Dialister* species or *Enterococcus* *faecalis* and tyramine^63,64^ (Spearman *ρ* > 0.54, *P* < 10^−10^, *q* < 0.1 for all; Fig. 3a and Extended Data Fig. 6a), as well as evidence for choline metabolism in *G.* *vaginalis*^65^ and *Corynebacterium* *aurimucosum*^66^ (*ρ* = 0.34, *P* < 10^−6^, *q* = 1.7 × 10^−5^ and *ρ* = 0.40, *P* = 4 × 10^−4^, *q* = 0.006, respectively). Additionally, higher tyramine concentrations were previously found in BV^67^, supporting the associations we found with BV-associated microbes (Fig. 3a).

We note that xenobiotics positively associated with sPTB have significantly weaker correlations with vaginal microbes than those observed for the rest of the metabolites (Mann–Whitney *P* = 0.024). DEA, for example, shows only weak correlations with all vaginal microbes (*ρ* < 0.23, *q* > 0.1 for all microbes). This observation provides further support for an exogenous source for these metabolites.

We found the strongest and most numerous correlations for tyramine (35 associations, Spearman 0.27 < *ρ* < 0.73; Fig. 3a), which was higher in TB among White women (Fig. 2a). Eight out of the 35 tyramine-correlated microbes are also correlated with choline, which was enriched in TB across all women (Fig. 2a). Interestingly, many of the species positively correlated with TB-associated metabolites, including *Atopobium* *vaginae, G.* *vaginalis*, several *Prevotella* species, BV-associated bacteria (BVAB) and many others, were previously reported to be associated with negative outcomes, such as BV^68^, PTB^13–15,17^ and other adverse pregnancy^69^ and neonatal^70^ outcomes. We found a similarly paradoxical negative correlation between *Staphylococcus* *epidermidis*, previously associated with BV^71^ and late-onset sepsis in preterm neonates^72^, and both tartrate and ethyl glucoside (*ρ* = −0.28, *P* = 6.9 × 10^−4^, *q* = 0.009 and *ρ* = −0.26, *P* = 0.0015, *q* = 0.016, respectively; Fig. 3a), which were positively associated with sPTB. Therefore, even as many of these associations were known, our results also suggest complex interactions among suboptimal vaginal microbes, sPTB-associated metabolites and health outcomes.

### Metabolic models support microbiome production of tyramine

**Extended Data Fig. 7:**
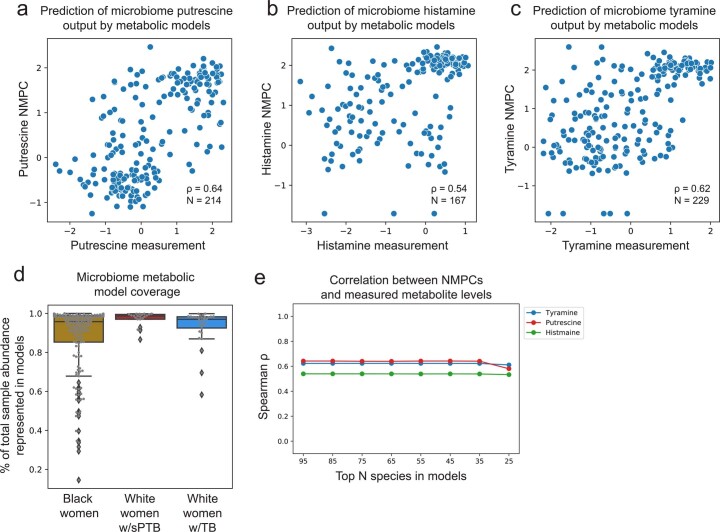
Metabolic models provide accurate predictions of putrescine, histamine, and tyramine. **a**–**c**, Putrescine (**a**), histamine (**b**), and tyramine (**c**) predictions derived from microbiome metabolic models (NMPC; Methods; y-axis) plotted against measured metabolite levels (x-axis), showing good accuracy for all (Spearman ρ = 0.64; ρ = 0.54; and ρ = 0.62, respectively, *P* < 10^−10^ for all). **d**, Model coverage (y-axis; line, median; box, IQR; whiskers, 1.5*IQR), described as the fraction of total sample abundance represented by metabolic models, for each subgroup separately. Samples from White women had higher model coverage compared to samples from Black women, despite the lower accuracy for tyramine prediction in the former group. N = 173 for Black women; N = 21 for White women with sPTB; N = 30 for White women with TB. **e**, Spearman ρ between metabolic model predictions (NMPCs) and metabolite measurements (y-axis) for models that only contain a maximum of N most abundant species (x-axis). As our metabolic models account for the abundance of each microbe, and as the vaginal microbiome has a skewed distribution, our models are robust to lack of representation of low-abundance microbes.

To gain some mechanistic insight into the correlations we found, we used community-level metabolic models^73^, which integrate genetic and biochemical knowledge to predict the metabolic output of each microbiome sample (community net maximal production capacity^73^ (NMPC); Methods). Our models show accurate predictions for several metabolites known to be produced by the vaginal microbiome^63,74^, such as putrescine and histamine (Spearman *ρ* = 0.64 between NMPCs and metabolomic measurements, *N* = 214, *P* < 10^−10^ and *ρ* = 0.54, *N* = 167, *P* < 10^−10^, respectively; Extended Data Fig. 7a,b).

Two sPTB-associated metabolites, tyramine and choline, were represented in our models. As our models predicted that choline was not affected by the vaginal microbiome (NMPCs of 0 for all women), we focused on tyramine, which previous studies suggest is produced by vaginal microbes^63,74^. Following genomic curation (Methods), the predictions of our models were highly accurate (Spearman *ρ* = 0.62, *N* = 229, *P* < 10^−10^; Extended Data Fig. 7c). Interestingly, we found that, among White women, while the measured levels of tyramine were enriched in TB (Mann–Whitney *P* = 2.8 × 10^−4^; Fig. 3b), its predicted microbiome output was not, and was even somewhat higher in sPTB (*P* = 0.26; Fig. 3c). This stems from lower accuracy in tyramine predictions in White women who delivered preterm (Spearman *ρ* = 0.19 versus *ρ* = 0.65, *P* = 0.02 for difference in ρ’s; Fig. 3d).

This difference in accuracy could not be explained by the representation of microbes in the metabolic models, which was in fact lower in Black women (Mann–Whitney *P* = 0.05, Extended Data Fig. 7d), probably due to the generally higher vaginal microbial diversity in this population^75^. Furthermore, tyramine prediction accuracy was not sensitive to constraints on metabolite uptakes or to the representation of low-abundance taxa (Methods, Supplementary Table 5 and Extended Data Fig. 7e). As these analyses suggest that lower tyramine prediction accuracy in White women with sPTB is not the result of a modelling artefact, the different accuracy could stem from a difference in strains, functional capacity or a non-microbial effect. Either phenomenon also has the potential to explain the aforementioned paradoxical microbial associations with tyramine (Fig. 3a). The possibility of a microbial difference or a host effect is also supported by AMON^37^, which predicts that tyramine is either microbial or host derived (Supplementary Table 3). Overall, our results demonstrate the utility of metabolic models in studying microbiome–metabolome interactions, and raise intriguing hypotheses for further investigation.

### Early prediction of sPTB risk using the vaginal metabolome

**Extended Data Fig. 8:**
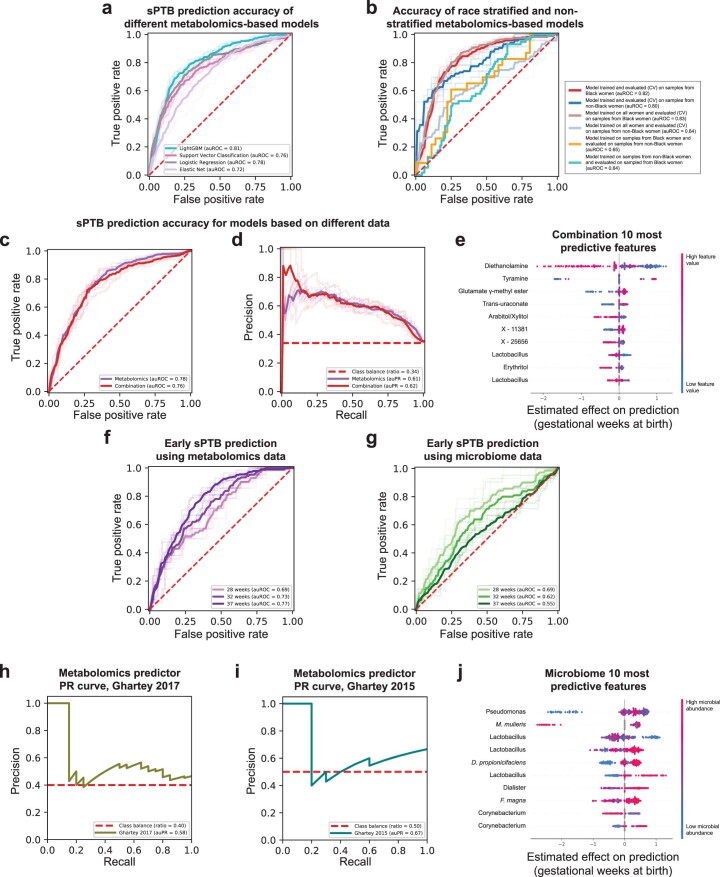
Performance and features of prediction models for sPTB. **a**, Receiver operating characteristic (ROC) curve comparing the performance of different sPTB prediction algorithms on metabolomics data. LightGBM (auROC = 0.81) outperforms logistic regression (auROC = 0.78, *P* = 0.017 for auROC comparison against LightGBM), support vector classification (auROC = 0.76, *P* = 2.9 × 10^−4^) and elastic net (auROC = 0.72, *P* = 0.004). **b**, ROC curve comparing the performance of a composite model stratified for race against a model trained on all samples. A model trained on samples from all women achieves the same accuracy as a model trained only on samples from Black women when evaluated in 10-fold cross-validation on sPTB prediction for Black women (auROC of 0.83 and 0.82, respectively). However, a model trained on samples from all women significantly underperforms a model trained only on samples from women who do not identify as Black when evaluated in 10-fold cross-validation on the same subgroup (auROC of 0.64 vs. 0.80, *P* = 4 × 10^−7^ for auROC comparison). Demonstrating that a different model is learned on each subgroup, models trained separately on each subgroup do not generalize as well to the other subgroup (auROC of 0.64 and 0.65). **c, d**, ROC (**c**) and precision-recall (PR; d) curves, evaluated in nested cross-validation, comparing sPTB prediction accuracy for models based on metabolomics data alone (auROC = 0.78, auPR = 0.61), and on metabolomics data combined with microbiome and clinical data (‘combination’; auROC = 0.76, auPR = 0.62; *P* = 0.44). **e**, SHAP^83^-based effect on total prediction (x-axis) for the top 10 features used in our combination models, sorted with descending importance. Each dot represents a sample, with the color corresponding to the metabolite level in the sample compared to all samples. **f, g**, ROC curves for the same metabolome-based (**f**) and microbiome-based (**g**) models as in Fig. 4a,b, when prediction is evaluated for extremely (<28 weeks of gestation) and very (<32 weeks) PTB. The microbiome-based models show increasing accuracy for predicting extremely and very PTB (auROC of 0.69 and 0.62, respectively, compared to auROC of 0.55 for all sPTB, *P* = 0.03 and *P* = 0.49, respectively). **h, i**, PR curve for sPTB prediction on two external cohorts, obtained using our metabolome-based predictor without retraining or adaptation. **j**, Same as (**e**) for the microbiome-based model. Shaded lines in **a–d, f, g** show results from five independent 10-fold cross validation draws (Methods). *p*-values for comparisons between ROC curves are based on the two-sided test described in ref. ^117^.

Early diagnosis of pregnancies with high risk for prematurity is crucial for the development of prevention and intervention strategies. We therefore explored whether we can use clinical, microbiome or metabolome data, collected ~3 months before delivery (mean ± s.d. of 14.5 ± 4.2 weeks), to predict subsequent sPTB. We used boosted decision trees, which were superior to alternative models (Extended Data Fig. 8a). For microbiome- and metabolome-based models, we trained composite predictors, such that a separate model was used for White and Black women. Despite the smaller effective sample size for each model, this resulted in better performance (Extended Data Fig. 8b). We evaluated all models on held-out samples using nested cross-validation without test data leakage (Methods).

**Fig. 4:**
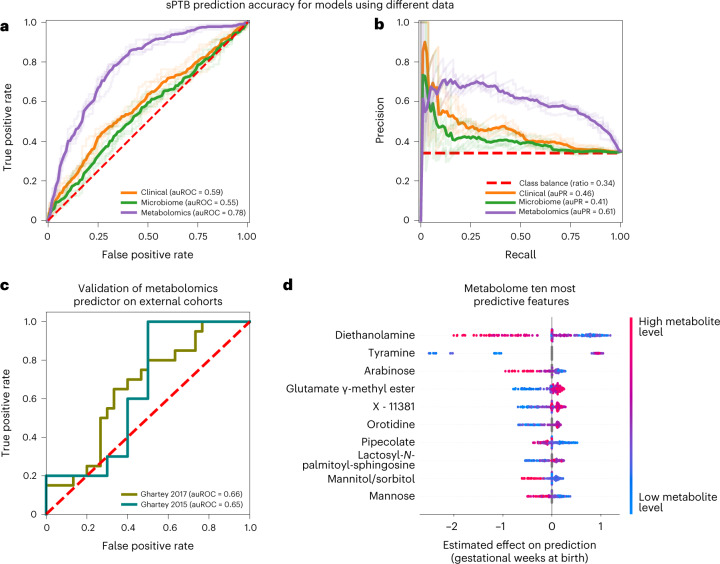
Metabolomics-based prediction of subsequent sPTB. **a**,**b**, Receiver operating characteristic (ROC, **a**) and precision-recall (PR, **b**) curves comparing sPTB prediction accuracy for models based on clinical (auROC = 0.59, auPR = 0.46), microbiome (auROC = 0.55, auPR = 0.41) and metabolomics (auROC = 0.78, auPR = 0.61) data (legend), evaluated in nested cross-validation (Methods). *N* = 232 for all. Shaded lines show results from five independent outer 10-fold cross-validation draws (Methods). **c**, ROC curve evaluating the performance of our metabolomics-based predictor on two external cohorts. Despite a challenging replication setting, with different inclusion criteria, measured metabolites and batch effects, our predictor obtains relatively accurate predictions without retraining (auROC = 0.66, auROC = 0.65, for the Ghartey 2017 (*N* = 50) and 2015 (*N* = 20) cohorts, respectively; Methods). **d**, Effect on total prediction (SHAP-based^83^; *X* axis) for the ten most predictive metabolites in our metabolome-based predictor, sorted with descending importance. Each dot represents a specific sample, with the colour corresponding to the relative level of the metabolite in the sample compared with all other samples.

Our models using clinical (age, body mass index (BMI), race, PTB history and nulliparity) and microbial abundance data, obtained limited accuracy (area under the receiver operating characteristic curve (auROC) of 0.59, area under the precision-recall curve (auPR) of 0.46 for clinical data; auROC = 0.55, auPR = 0.41 for microbiome data; *P* = 0.12 for difference between the models; Methods and Fig. 4a,b). Notably, using metabolomics data, we were able to generate a model with superior accuracy (auROC = 0.78, auPR = 0.61, *P* < 10^−10^ for comparison with either clinical or microbiome models; Methods and Fig. 4a,b). Lastly, a model combining clinical, microbiome and metabolomics data obtained similar accuracy to the metabolome-based model (auROC = 0.76, auPR = 0.62, *P* = 0.44 versus metabolome-based model; Extended Data Fig. 8c,d), with metabolites as the most prominent contributors to the model (Extended Data Fig. 8e). This suggests that metabolite measurements are a sufficient representation of information contained in these three data types with respect to sPTB.

Our metabolome-based model is superior or similar in accuracy to several previously published models, such as those using amniotic fluid metabolomics (auROC 0.65–0.70, *N* = 24) (ref. ^76^), maternal serum metabolome and clinical data (auROC 0.73, *N* = 164) (ref. ^77^), maternal urine and plasma metabolome (auROC 0.69–0.79, *N* = 146) (ref. ^78^), blood cell-free RNA measurements (auROC 0.81, *N* = 38) (ref. ^79^) or vaginal protein biomarkers (auROC 0.86, *N* = 150, sPTB *N* = 11) (ref. ^80^), many of which have small sample sizes, lack demographic diversity or focus on high-risk cohorts. Overall, our results demonstrate the promising utility of vaginal metabolites as early and accurate biomarkers of sPTB.

We next evaluated the same models, without retraining, for predicting extremely and very PTB in Black women from the same held-out data (that is, only the ground-truth classification of outcome changed). Interestingly, while the metabolome-based model showed a slight decrease in accuracy (auROC of 0.69 and 0.73 for extremely and very PTB, respectively, compared with auROC of 0.77 for sPTB in Black women; *P* = 4.3 × 10^−4^ and *P* = 0.001, respectively; Extended Data Fig. 8f), our microbiome-based model showed increasing accuracy (auROC of 0.69 and 0.62, respectively, compared with auROC of 0.55; *P* = 0.031 and *P* = 0.49, respectively; Extended Data Fig. 8g). These results may reflect the potentially increased involvement of the vaginal microbiome in earlier sPTBs^1^.

### Metabolome-based predictor replicates in external cohorts

To test the generalizability of our metabolome-based model, we validated its accuracy in two independent cohorts (Methods): a case–control study of 20 women (10 PTB), mostly (75%) White, at high risk for PTB, with samples collected at 24–28 weeks of gestation (‘Ghartey, 2015’) (ref. ^81^); and a case–control study of 50 women (20 PTB), mostly (88%) Black, presenting with symptoms of preterm labour and no PTB history, with samples collected at 22–34 weeks of gestation (‘Ghartey, 2017’) (ref. ^55^).

This validation was extremely challenging: due to the different inclusion criteria and population structure, substantial batch effects in metabolomics measurements across different studies^82^ and finally, as data were generated 4–6 years earlier, only a small fraction of metabolites used by our predictor were measured (34% and 39%). To emphasize this, only one and two (for Ghartey 2015 and 2017, respectively) of the ten associations we detected between vaginal metabolites and sPTB (Fig. 2a) could be examined in these cohorts (Methods), of which none were significant (Mann–Whitney *P* > 0.05). These sPTB-associated metabolites are probably important features for prediction, making generalization across these cohorts difficult. Despite this challenging setting, our metabolome-based predictor, trained only on the 232 samples profiled here, without any retraining or adaptation, provided relatively accurate predictions in both external cohorts (auROC = 0.65, auPR = 0.67 and auROC = 0.66, auPR = 0.58 for Ghartey 2015 and 2017, respectively; Fig. 4c and Extended Data Fig. 8h,i). These results demonstrate the robustness of the vaginal metabolome and of our predictive approach to study specific biases.

### Model interpretation reveals other contributing features

To obtain insights into the features used by the models, we assessed the contribution of each feature towards the prediction for each sample using SHapley Additive exPlanations (SHAP)^83^ (Supplementary Table 6). As expected, six of the ten most predictive metabolites, namely DEA, tyramine, arabinose, glutamate γ-methyl ester, mannitol/sorbitol and mannose, were also identified in our association analysis, with a similar direction of association (Figs. 2 and 4d). We additionally found that high pipecolate levels and low levels of lactosyl-*N*-palmitoyl-sphingosine and orotidine contribute to sPTB predictions. Of these, pipecolate was shown to be elevated in women with BV^84^.

A similar analysis of our microbiome-based predictor also captured previously detected associations between vaginal microbes and sPTB, including those of *Mobiluncus* *mulieris*^14^ and *Finegoldia* *magna*^85^, and of *Lactobacillus*^14^ and *Dialister* species^15^ (Extended Data Fig. 8j). These results highlight the interpretability of our models and their ability to model complex non-linear interactions, enabling us to expose associations not detected by univariate analyses.

## Discussion

In this study, we measured the second-trimester vaginal metabolome of 232 pregnant women. We show that it is associated with the vaginal microbiome, and that metabolite signatures are enriched for sPTB among Black women. We identify multiple metabolites that are associated with sPTB, across the cohort and separately for Black and White women. Our results highlight exogenous metabolites with strong associations with sPTB, which we suggest constitute important risk factors. We further uncover intriguing interactions between TB-associated metabolites and potentially suboptimal microbes, and propose a difference in the vaginal metabolism of tyramine in White women who delivered preterm. Finally, we demonstrate that metabolome-based models can predict subsequent sPTB weeks to months in advance, potentially paving the way for early diagnostics.

We detected several sPTB-associated xenobiotics: DEA, ethyl glucoside, tartrate and EDTA, which prior literature and a functional analysis^37^ suggest are of exogenous source. DEA, a chemical with no known natural source^86^, commonly used in drilling and metalworking fluids^35^, and to which reproductive-aged women are highly exposed^87^, and ethyl glucoside, present in alcohol-containing products^31^, are both precursors or ingredients in hygienic and cosmetic products^30,33^. Tartrate and EDTA are used as food additives^32,58^ and are also common in hygienic and cosmetic products^32,57^. While we have not identified the sources of these metabolites, the fact that all are documented in hygienic and cosmetic products raises concern that some of these products may increase the risk of sPTB. Our results coincide with recent studies raising concerns regarding environmental exposures in pregnancy^88,89^, and identify these chemicals in the reproductive tract. Further study is warranted to identify the sources of these metabolites and to disentangle their effects on the host, microbiome and pregnancy outcomes, so that policy recommendations can be made regarding their use in various products and during pregnancy.

The cohort we analysed included a majority of Black women, offering an opportunity to study PTB in women who are disproportionately burdened by PTB and other adverse pregnancy outcomes, while also represented in small numbers in many studies. However, we urge caution in drawing conclusions from differences in associations between Black and White women, as maternal self-identified race represents a complex array of pre-existing differences, disparities and clinical covariates at the time of sampling. Nevertheless, we note that the enrichment of sPTB associations among the xenobiotic metabolite set in Black women may potentially reflect disparities in environmental and exogenous exposures^90,91^, consistent with reports that Black women have greater exposures to endocrine disrupting chemicals through personal care products^92,93^ and with studies that identified exogenous chemicals as possible drivers of PTB^94,95^. Metabolomic exposure patterns could contribute to the association between racial disparities in prematurity rates and racial differences in the vaginal microbiome^96^.

We used community-scale metabolic models to investigate microbial tyramine metabolism, which have important limitations. Model curation is an ongoing effort, and thus models may not be tailored to each sample or may lack representation of niche-specific metabolic capabilities. Another limitation stems from the resolution of 16S rRNA amplicon sequencing, which identifies taxa at the species or genus level, precluding strain-specific modelling. Despite these limitations, our models accurately predicted several metabolites, and offered insights regarding potential sources of tyramine.

Our predictive modelling approach has several noteworthy limitations: (1) our use of a case–control cohort enriched for sPTB limits our ability to assess population-level predictive value, and further validation is required in prospective studies. (2) As this cohort was focused on sPTB, we are unable to assess if our models are specific to sPTB or are detecting a general risk for adverse pregnancy outcomes. (3) The use of race in our models, while common throughout medicine^97^, is controversial and creates issues in implementation^98^. This was driven by differences in both sample size and the vaginal metabolome itself between Black and White women in this cohort, and resulted in an overall increased accuracy. (4) Finally, there is additional unexplored potential in using even earlier samples for prediction. A larger sample size, and combination with other sources of data, such as maternal urine or serum metabolomics, vaginal metagenomics or cell-free RNA measurements, could further improve prediction accuracy.

Our results demonstrate the utility of vaginal metabolites as early biomarkers of PTB, and identify xenobiotic metabolites as potentially modifiable sPTB risk factors, which may also disproportionately affect Black women. The strong associations we observe motivate the investigation of the vaginal microbiome and metabolome in the context of other adverse pregnancy outcomes such as pre-eclampsia, indicated PTB and BV.

## Methods

### Study design and cohort description

We analysed banked samples from the previously collected and described Motherhood and Microbiome cohort (NCT02030106) (ref. ^14^). This cohort was approved by the institutional review board at the University of Pennsylvania (IRB 818914) and the University of Maryland School of Medicine (HP-00045398), and all participants provided written informed consent. The Motherhood and Microbiome cohort recruited 2,000 women with a singleton pregnancy before 20 weeks of gestation. Women were followed to delivery, and sPTB was defined as delivery before 37 weeks of gestation with a presentation of cervical dilation and/or premature rupture of membranes. Of these, the vaginal microbiota of 503 women was previously characterized via 16S rRNA gene amplicon sequencing (V3–V4 region) of vaginal swabs collected between 20 and 24 weeks of gestation, and total bacterial load was assessed using the TaqMan BactQuant assay^14^. For this study, out of women with available microbiome data, all available samples were selected from women who delivered preterm (*N* = 80), in addition to samples from 152 controls who delivered at term. The selected cervicovaginal samples were replicates of those used for 16S rRNA gene sequencing, collected using a double shaft dacron swab. Cervicovaginal swabs were either self-collected or collected by a research coordinator during a study visit^14^.

### Statistics and reproducibility

No data was excluded from analysis in the present study. As the study was observational, there was no allocation or randomization. The study included all available samples who delivered preterm (*N* = 80), and no statistical methods were used to pre-determine sample sizes; our sample size is similar to those reported in previous publications^25,26^. Samples were randomly distributed across metabolomics batches and metabolomics analysis was performed by Metabolon, who were blinded to the outcome assessment of each sample. Two-sided Mann–Whitney *U* tests (SciPy 1.5.2) and logistic regression (Statsmodels 0.12.1) were used to identify associations between metabolite levels and sPTB. Two-sided Fisher’s exact tests (R stats 3.6.1) were used to identify associations among MCs, CSTs, race and sPTB. PERMANOVA tests (scikit-bio 0.5.6) were used to identify associations among the microbiome, metabolome, CST, race and metabolomics batches. Metabolite set enrichment analysis (Methods) was used to identify associations between metabolite sets and sPTB. Spearman correlations were used to measure the agreement between metabolite levels and NMPCs and between metabolite levels and microbial abundances. Fisher *R*-to-*z* transform was used to compare correlations measured within subgroups. Evaluation of machine learning models was performed using scikit-learn 0.24.2. pandas 1.1.5 and NumPy 1.18.5 were used for data processing. Robust assessment of generalization error of predictive models was achieved via nested cross-validation.

### Metabolomics profiling and preprocessing

Metabolite levels were measured from vaginal swabs by Metabolon, using an untargeted liquid chromatography–tandem mass spectrometry (LC-MS/MS) platform^99^. For discussion of batch processing of the sample, see Supplementary Note 1 and Extended Data Fig. 2. We note that swab lot number, sterile swabs for blank processing and sample collector (coordinator or self-collection) are not available. While this limits analysis of potential batch effects, we find batch confounding (for example, swab lot associated with sPTB) unlikely as samples were collected before delivery and outcome determination.

Following a methanol-based small-molecule extraction, samples were divided into 5 µl aliquots and each was resuspended in an appropriate extraction solvent and separated via one of four chromatography techniques. Each chromatographic method was optimized for the extraction of hydrophobic, basic or polar compounds. The chromatographic method used for the quantification of each metabolite is provided in Supplementary Table 4. Isotopically labelled or halogenated standards were added to all aliquots at fixed concentrations before extraction to serve as retention time markers. Following extraction, compounds were subjected to electrospray ionization and measured via tandem mass spectrometry by a Q-Exactive Hybrid Quadrupole-Orbitrap high resolution mass spectrometer. Data-dependent acquisition mode was used to generate fragmentation spectra of high-intensity *m*/*z* peaks detected during the first round of mass spectrometry. *m*/*z* peaks were identified and annotated by Metabolon using proprietary software and comparisons to their database of retention indices and fragment ion spectra. The areas under annotated *m*/*z* peaks were taken as metabolite measurements. A comprehensive overview of all chromatographic and mass spectrometry parameters is available in Supplementary Table 7. Process blanks (negative controls) were run with each metabolomic plate, and metabolites were considered present only if they were detected with levels that were at least three times higher than these controls. Detected levels of the xenobiotics highlighted in this study, in vaginal samples and negative controls, are shown in Extended Data Fig. 5e, demonstrating the same. For the mass error of these xenobiotics, see also Extended Data Fig. 5f, showing high identification quality compared with other non-xenobiotic metabolites.

While the majority of named metabolites (*N* = 556) were tier 1 identified by Metabolon via fragmentation spectra matches to experimentally measured library standards, only tier 2 assignments are available for independent identification due to the proprietary nature of the Metabolon platform. Metabolite measurements were volume normalized to the volume of buffer used, which may not necessarily account for differences in the original tissue. This was followed by robust standardization^27^ of the log (base 10) transformed values (subtracting the median and dividing by the standard deviation calculated while clipping the top and bottom 5% of outliers). The Shapiro–Wilk test was used to determine that log (base 10) transformed values deviated from normality for the majority of metabolites (389 of 635 named metabolites). For this reason, non-parametric tests were used in subsequent metabolomic analyses.

### Microbiome data processing

All microbiome-based analyses were done using data previously processed with DADA2 (ref. ^100^) and SpeciateIT^14^, available from Supplementary Data 2 of ref. ^14^. A single exception to this are predictive models, which were trained on 97% clustered operational taxonomic units (OTUs) using the USEARCH pipeline^101^. We obtained raw sequences from the database of Genotypes and Phenotypes (dbGaP) under study accession: phs001739.v1.p1. Primers were aligned to reads and then trimmed, followed by end merging and quality filtering (-fastq_maxee 1.0). The filtered reads were then pooled together, dereplicated, clustered with a 97% threshold and chimera filtered with the UPARSE algorithm to produce the OTU count matrix.

### Global microbiome and metabolome structure

PERMANOVA analysis was performed using Bray–Curtis distance for microbiome data and the Canberra distance for metabolites data, which is robust to outliers and sensitive to differences in common features. De novo clustering of metabolite vectors was done using the *k*-medoids algorithm (scikit-learn-extra 0.2.0), also with the Canberra distance. We determined the optimal number of clusters by comparing the within cluster sum of square error and the gap statistic for clustering solutions with *k* between 1 and 15 (Extended Data Fig. 3a,b). To check the robustness and consistency of these clusters, we performed 100 random selections of 209 (90%) of the 232 samples, recreating clusters de novo with the same procedure for each random subset. Many of the resulting subsets (36) had over 95% of samples assigned to the same metabolite cluster as the original assignment (Supplementary Table 2), with an average assignment accuracy of 86% across all random subsets (Extended Data Fig. 3g), demonstrating that our metabolite clusters are indeed consistent. Uniform manifold approximation and projection (UMAP)^102^ was performed using the Python umap-learn package^102^, with n_neighbors of 15 and min_dist of 0.05 for microbiome data and n_neighbors of 15 and min_dist of 0.25 for metabolomics data. To further describe each metabolomics cluster, Fisher’s exact test was used to identify metabolite super and subpathways enriched among metabolites associated with each cluster (*P* < 0.05).

### Differential abundance testing and metabolite set enrichment analysis

Differential abundance tests between metabolite levels were done using the two-sided Mann–Whitney *U* test for metabolites that were present in at least half of the cases. All associations with early PTB were calculated using only samples from Black women, due to their high proportion among these deliveries (21 of 26 for childbirths <32 weeks of gestation and 14 of 15 for childbirth <28 weeks). To identify functional sets of metabolites that were perturbed between sPTB and TB, we compared, for each set, the Mann–Whitney *P* values for differential abundance between PTB and sPTB for metabolites within the set to the same *P* values for metabolites outside the sets, using an additional Mann–Whitney *U* test. We calculated significance by comparing the *P* value of the latter test to 10,000 similar *P* values calculated on random permutations of sPTB and TB labels. For functional sets, we used definitions of super and subpathways provided by Metabolon, as well as KEGG^61^ pathways. False discovery rate (FDR) correction was performed separately for each metabolite set type.

### Prediction of metabolite origins using AMON

AMON^37^ is a method that uses functional annotations according to the KEGG database^61^ to predict metabolite origins for all metabolites that could be matched to a KEGG entry (*N* = 334 of 635 named metabolites). We used PICRUSt2 (ref. ^103^) to generate functional profiles for each sample, and then applied AMON^37^ to predict whether metabolites that had matching entries in the KEGG Database are products of human or microbial metabolism. When both were false, we interpreted the metabolite to be a xenobiotic.

### Microbe–metabolite correlations

To identify associations between microbes and metabolites, we estimated microbial absolute abundance by multiplying the relative abundances of each taxon by the total 16S rRNA copy number for the sample, obtained using the TaqMan quantitative polymerase chain reaction (qPCR)-based panel^14,104,105^, and calculated Spearman correlations with the levels of metabolites we found to be associated with sPTB. Across all correlation network analyses (Fig. 3a and Extended Data Figs. 6a,c–e) we included correlations with at least 22% of paired measurements, corresponding to 50 samples of 232 for Fig. 3a. All correlation measurements used available data without imputation, and correction for multiple testing was performed via the Benjamini–Hochberg FDR method. To determine whether edges in our network were influenced by race (Extended Data Fig. 6b) or by the severity of sPTB (Extended Data Fig. 6f), we used a two-sided Fisher *R*-to-*z* transform to compare these correlations in Black women to the same correlations in White women, as well as to compare these correlations in Black women who delivered before 32 weeks to the same correlations in all other Black women.

### Creating and interrogating vaginal microbiome models

Microbiome metabolic modelling was done using Microbiome Modeling Toolbox (COBRA toolbox commit: 71c117305231f77a0292856e292b95ab32040711) (refs. ^73,106^), using models from AGORA2 (ref. ^107^). All computations were performed in MATLAB version 2019a (Mathworks), using the IBM CPLEX (IBM) 12.10.0 solver.

For each sample, tailored microbiome models were created through the compartmentalization technique^108^: metabolic reconstructions of species present in the sample are merged into a shared compartment, and input and output compartments are added. The shared compartment enables microbes to share metabolites while input and output compartments are present to enable compounds intake and secretion. Coupling constraints are added as in refs. ^109,110^ to ensure a dependency between relative abundances and each species network fluxes. Finally, sample-specific microbiome biomass objective functions, composed by the sum of each microbial biomass multiplied by the corresponding relative abundance value, are added to each microbiome model.

To interrogate the secretion potential of each sample-specific microbiome model, we computed NMPCs using the pipeline mgPipe.m of the Microbiome Modeling Toolbox^73^ (Supplementary Table 8). NMPC calculation accounts for maximal microbiome compound production and uptake rates, and aims at predicting the overall contribution of microbiomes to the metabolism of specific compounds^73^. To later assess prediction accuracy, we computed Spearman correlations between NMPCs and the corresponding metabolite measurements without imputation.

To support and improve the accuracy of our tyramine predictions, we validated the presence of the *TDC* gene, coding for tyrosine decarboxylase. For each species represented in our metabolic models (*N* = 95), we used Prodigal^111^ to predict open reading frames in up to 200 randomly selected Refseq^112^ assemblies, and searched them for evidence of *TDC* using the hmmsearch function of Hmmer3.3.2 (ref. ^113^) and a profile hmm for *TDC*^114^ (NCBI HMM accession TIGR03811.1). We then curated our metabolic models, making sure that the corresponding reaction exists in models for which at least one assembly contained the corresponding gene.

To compile the metabolic models, we matched between the species detected in the microbiome samples and those present in AGORA2 (ref. ^107^) (Supplementary Table 9). To increase the representativeness of our models, we added three representatives for abundant vaginal species without a corresponding AGORA2 model that were present with >5% relative abundance in at least 20 samples (listed in Supplementary Table 9). The only species that passed this threshold, which was not included in our models was *Candidatus* Lachnocurva vaginae (BVAB1), for which no suitable AGORA model was available. To generate species-level models, we combined metabolic models from available strains using the function createPanModels.m of the Microbiome Modeling Toolbox^73^. Altogether, our microbiome metabolic models included 95 different species, with an average of 20 species in each sample. As the vaginal microbiome has a very skewed distribution^28^, this resulted in a median (interquartile range (IQR)) of 96.7% (88.4–98.8%) of the total abundance across samples represented by our models (Extended Data Fig. 7d).

As a test of the sensitivity of our models to the lack of representation of low-abundance microbes, we performed simulations where we iteratively removed the ten least abundant species from consideration by our models, and evaluated the accuracy of our models in predicting the well-modelled metabolites tyramine, putrescine and histamine. As expected, as our models account for the abundance of each microbe, and as the vaginal microbiome has a skewed distribution, our models were not sensitive to the representation of low-abundance microbes (Extended Data Fig. 7e), even when removing 70 out of 95 models.

Metabolic modelling requires environmental conditions such as media and carbon source availability^115^. We therefore formulated a ‘general vaginal media’ (Supplementary Table 10), as the union of all metabolites present in at least 50 samples to which a corresponding metabolite was identified in AGORA, assuming them to be present in an unlimited (that is, very high) concentration. This vaginal media was applied to each microbiome model input compartment in the form of constraints on metabolite uptake reactions, constraining uptake of compounds not present in the environment to zero. Uptake of specific gut-related dietary compounds, automatically performed in mgPipe, was disabled acknowledging the different metabolic environment in the vagina, and essential metabolites required for achieving microbiome growth, together with their respective flux value, were detected and added to the vaginal media using the fastFVA and findMIIS functions of the COBRA toolbox^106^. A comparison of the ‘general’ media to subgroup-specific media, defined as metabolites present in 75% of samples from Black and White women separately, with uptake fluxes constrained to the mean value across the subgroup, and to a person-specific media, in which uptake fluxes were constrained for each sample separately, showed similar accuracy with respect to tyramine predictions (Supplementary Table 5).

### Training, testing and validation of sPTB classifiers

We constructed predictive models separately using the clinical (age, race, parity status, history of sPTB and BMI), microbiome and metabolomics data, as well as a combination model consisting of all of these data types combined. As race had very strong interactions with microbiome and metabolomics data, we trained a composite predictor for microbiome, metabolomics and combination models, whereas a separate model was trained for Black women. Despite the smaller sample size for each model, this empirically improved prediction performance (Extended Data Fig. 8b). Microbiome-based models used absolute abundances, calculated from USEARCH-processed OTUs as described above. In cases where qPCR-based total load was not available (*N* = 14), it was imputed to the mean total load using only training samples.

Samples were split into training and test sets using 10-fold cross-validation (‘outer folds’), block-stratified for deciles of gestational age at birth (GAB), and for microbiome, metabolomics and combined models, also stratified for race. To account for stochasticity in the division to ten folds, we repeated this process five times. Train–test sterility was strictly maintained. To tune the optimal set of hyperparameters (including parameters for feature engineering and selection), and to obtain a robust estimate of the generalization error, we used nested cross-validation. In this extension of the training–test–validation framework, the training set was further split to five folds (‘inner folds’), on which we used 1,000 iterations of a random set of hyperparameters (Supplementary Table 11). Once more, to account for stochasticity, we repeated this process five times. We selected the best hyperparameter set as the model with the top average auROC score out of the top five most accurate models based on average *R*^2^ for sPTB classification, based on performance on the inner folds. We then used these hyperparameters to train a model on the entire training data for the outer fold, and evaluated it on the held-out test data. Of note, in this framework, hyperparameters are selected using strictly the training data of each outer 10-fold cross-validation fold, and are evaluated just once on the test set. Our prediction pipeline included standardization and imputation (for metabolomics data), optional principal component analysis (PCA) transformation, and feature selection using sparsity, SHAP^83^ feature importance, information gain and/or Spearman correlation, followed by prediction using LightGBM^116^, with all steps performed strictly using training data. The selected models were then evaluated, without retraining, on classification of extremely (GAB <28 weeks) or very (GAB <32 weeks) PTB on the outer fold. Benchmark analyses (Extended Data Fig. 8a,b) were done using 10-fold cross-validation, repeated five times. We assessed the significance of the difference in auROC between two models by computing *z*-scores of the normal distributions of auROCs^117^.

To obtain a final model for interpretation and validation, we trained new composite models on the entire cohort (*N* = 232), using the hyperparameters selected for each of the outer folds (50 models), and picked the model with the best auROC on the same cohort (training fit). The final parameter set for each model is listed in Supplementary Table 12. For validation on external vaginal metabolome datasets, we note that information on maternal race at the subject level was not available to us. We therefore applied the metabolomics model used for non-Black women, without retraining or adaptation, to metabolomics data from the Ghartey 2015 (ref. ^81^) cohort, as this cohort contained mostly White women; and similarly applied the metabolomics model used for Black women to metabolomics data from the Ghartey 2017 (ref. ^55^) cohort. For validation of associations of metabolites with sPTB (Fig. 2a) in these cohorts, we note that, of the ten metabolites in Fig. 2a, only the six that apply to all and White women can be validated in the Ghartey 2015 cohort, of which only one was measured; and only the nine that apply to all and Black women can be validated in the Ghartey 2017 cohort, of which only two were measured.

### Reporting summary

Further information on research design is available in the Nature Portfolio Reporting Summary linked to this article.

## Supplementary information

Supplementary Information Supplementary Notes 1–2.

Reporting Summary

Supplementary Table **Supplementary Table 1** **Raw metabolite measurements**. Sample IDs refer to Supplementary Data 2 of ref. ^14^. Values are raw area counts. **Supplementary Table 2** **Assignments of samples to metabolite clusters (MCs)**. Sample IDs refer to Supplementary Data 2 of ref. ^14^. **Supplementary Table 3** **Metabolite origin predictions by AMON. Supplementary Table 4** **Metabolite annotations and extraction platforms. Supplementary Table 5** **Tyramine prediction accuracy with metabolic models using different media definitions**. Values are Spearman *ρ* between NMPC values and tyramine measurements. **Supplementary Table 6** **Shapley values of prediction models. Supplementary Table 7** **Chromatography and mass spectrometry parameters**. Listed are all technical parameters for each of Metabolon’s LC–MS/MS platforms. **Supplementary Table 8** **Tyramine, putrescine and histamine predicted NMPCs**. Sample IDs refer to Supplementary Data 2 of ref. ^14^. **Supplementary Table 9** **Assignments of SpeciateIT species to AGORA models**. SpeciateIT species are the columns of Supplementary Data 2 of ref. ^14^. **Supplementary Table 10** **Metabolites included in the vaginal media used in metabolic models**. Listed are the metabolites included, along with their AGORA identifiers. **Supplementary Table 11** **Hyperparameter sets used to optimize prediction models. Supplementary Table 12** **Parameters of final prediction models. Supplementary Table 13** **Measurement characteristics for highlighted xenobiotics.**

## Data Availability

The 16S rRNA gene amplicon sequencing data and the associated samples and subjects’ metadata analysed in this study are publicly available in the database of Genotypes and Phenotypes (dbGaP) under accession number phs001739.v1.p1 as well as in Supplementary Data 2 of ref. ^14^. Raw metabolomics data are available in Supplementary Table 1. Mass spectral data are available from MetaboLights under accession number MTBLS702 (https://www.ebi.ac.uk/metabolights/MTBLS702). Additional information regarding xenobiotics is provided in Supplementary Table 13. The KEGG Database is available at https://www.genome.jp/kegg/, and the AGORA models are available at https://www.vmh.life/.

## Code availability

Scripts to reproduce the analysis are available in a GitHub repository: https://github.com/korem-lab/PTB_Metabs_2021. The mgPipe pipeline is available within the COBRA toolbox (https://github.com/opencobra/cobratoolbox).
