# Supplementary material for: Preterm birth is associated with xenobiotics and predicted by the vaginal metabolome
Source: Nat Microbiol. 2023 Jan 12;8(2):246–59. doi: 10.1038/s41564-022-01293-8 (PMC9894755; doi:10.1038/s41564-022-01293-8)
Supplement: Supplementary file 1 — Supplementary Notes 1–2. [file 41564_2022_1293_MOESM1_ESM.pdf]

nature microbiology

Article

https://doi.org/10.1038/s41564-022-01293-8

Preterm birth is associated with xenobiotics and predicted by the vaginal metabolome

In the format provided by the authors and unedited

Supplementary notes

Supplementary Note 1: The effect of processing batches on our analyses

Due to the size of this cohort, metabolomics measurements had to be split across a few days. For three of the four metabolomics platforms used (LC MS/MS Pos Early, Pos Late, and Polar), samples were analyzed in two batches (batch 1, N=114; batch 2, N=118). For the fourth platform (Neg), batch 1 was split across two days (batch 1, N=83; batch 3, N=31). Differences between batches are to be expected[82], and we indeed detect some changes between our different batches (PERMANOVA on Canberra distance, p=0.09 and p=0.02 for two and three batches, respectively; **Extended Data Fig. 2a,b**). We note, however, that neither of the larger two batches are significantly separated from the rest of the cohort samples in the three batch structure (p=0.18 and p=0.08).

To demonstrate that results across our study are robust to this two- or three-batches structure, we have performed several benchmarks and validation analyses. First, we show that none of the MCs which we identified are enriched for any particular batch (Fisher's exact p=0.93 and p=0.93 for two and three batches; **Extended Data Fig. 2c**).

To determine whether the associations of specific metabolites with sPTB were influenced by batch effects, we reanalyzed all metabolites that were significantly associated with sPTB (**Fig. 2a**), using logistic regression to model the effect of metabolite levels on sPTB risk while adjusting for batch, using the appropriate batch assignment according to the platform of each metabolite (**Supplementary Table 4**). Notably, all associations that were significant in our association analysis (**Fig. 2a**) were also identified as significantly associated with analysis when adjusting for batch structure (FDR < 0.1; **Extended Data Fig. 2d**). This analysis demonstrates that the associations we detected between metabolites and sPTB are robust to batch effects.

We then investigated whether the accuracy of our metabolite based predictive model is a result of our model learning a signal generated by variations between batches. We trained predictive models in a similar fashion to the models developed on the entire data (Methods), but in 10-fold cross-validation limited to samples from either of the two batches. These batch-specific models showed reduced accuracy compared to models using the entire data (auROC of 0.66 for both models; **Extended Data Fig. 2e**). To show that this reduction in accuracy is due to sample size, we randomly sampled a similar number of samples (N=116) from the entire cohort, and repeated the analysis 50 times, resulting in an average auROC of 0.67 (**Extended Data Fig. 2e**; p=0.44 for both batch 1 and 2, for rejecting the null hypothesis that the batch-specific model accuracy is lower than expected). We next checked if the batch-specific models generalize to the other batch, by training a final batch-specific model on all samples from one of the batches and validating it on samples from the other batch without any adjustments or retraining. Both batch-specific models showed similar generalization accuracy (auROC of 0.66 for both the batch 1 model evaluated on batch 1 samples in cross validation and on batch 2 samples; and auROC of 0.66 vs. 0.69 for the batch 2 model similarly evaluated on batch 2 and batch 1, respectively; **Extended Data Fig. 2e**). These results demonstrate that our metabolomics-based predictor is also robust to batch effects in the metabolomics data.

Supplementary Note 2: Interaction of microbe-metabolite associations with race and sPTB timing.

As many of the associations between metabolites and sPTB were modulated by race, we investigated whether the associations in our microbe-metabolites correlations network (**Fig. 3a**) are likewise influenced. We find that nine of the 68 microbe-metabolite associations we detected were significantly different (Fisher's R-to-z $p < 0.05$; Methods) between Black and White women, although a different direction of association was detected in only four of these (**Extended Data Fig. 6b**; see also **Extended Data Fig. 6c,d** for subgroup-specific correlation networks). Specifically, *G. vaginalis, A. vaginae*, and three other species that were positively associated with tyramine, had significantly stronger associations in Black women ($p < 0.02$ for all). Recent studies show that biofilm interactions between *G. vaginalis* and other microbes, including *A. vaginae*[118,119], may contribute to BV, suggesting that the differences in tyramine associations between Black and White women may be related to differences in community structure and microbial interactions. Taken together, however, we find a relatively small effect of race on microbe-metabolite correlations.

Since there is evidence that the vaginal microbiome may be more strongly associated with earlier sPTB[1], we investigated whether vaginal microbes were correlated with metabolites associated with these earlier sPTBs in samples from Black women (**Fig 2d, Methods**). We identified significant associations between only two microbes, *Anaerococcus prevotii* and *Shigella flexneri*, and one metabolite, EDTA, which was elevated in early PTB (**Fig. 2d**). Both *A. prevotii* and *S. flexneri* were negatively associated with EDTA (ρ=-0.37, p=5.1x10^{-5} and ρ=-0.37, p=0.0002, respectively, **Extended Data Fig. 6e**). *S. flexneri* has been previously associated with both vaginitis and preterm labor[120], and its correlation with an early-PTB-associated metabolite may reflect the increased incidence of reproductive tract infections in earlier PTBs[1]. Next, we investigated whether the vaginal microbiome of women who subsequently had early sPTB has different associations with sPTB-associated metabolites (**Extended Data Fig. 6f**). We find only one association, the negative correlation between *Ca. L. vaginae* (BVAB1) and glutamate gamma methyl-ester, which was significantly stronger in subjects who subsequently had early PTB. Overall, we find a relatively small effect of sPTB timing on microbe-metabolite correlations.

Supplementary references

118. Castro, J., Machado, D. & Cerca, N. Unveiling the role of *Gardnerella vaginalis* in polymicrobial bacterial vaginosis biofilms: the impact of other vaginal pathogens living as neighbors. *ISME J.* **13**, 1306-1317 (2019).
119. Castro, J., Rosca, A. S., Cools, P., Vaneechoutte, M. & Cerca, N. *Gardnerella vaginalis* enhances *Atopobium vaginae* viability in an in vitro model. *Front. Cell. Infect. Microbiol.* **10**, 83 (2020).
120. Kotloff, K. L., Riddle, M. S., Platts-Mills, J. A., Pavlinac, P. & Zaidi, A. K. M. Shigellosis. *Lancet* **391**, 801-812 (2018).
