# Supplementary material for: Preterm birth is associated with xenobiotics and predicted by the vaginal metabolome
Source: Nat Microbiol. 2023 Jan 12;8(2):246–59. doi: 10.1038/s41564-022-01293-8 (PMC9894755; doi:10.1038/s41564-022-01293-8)
Supplement: Supplementary file 2 — Reporting Summary [file 41564_2022_1293_MOESM2_ESM.pdf]

nature portfolio

Corresponding author(s): Maayan Levy, Tal Korem

Last updated by author(s): Nov 18, 2022

Reporting Summary

Nature Portfolio wishes to improve the reproducibility of the work that we publish. This form provides structure for consistency and transparency in reporting. For further information on Nature Portfolio policies, see our Editorial Policies and the Editorial Policy Checklist.

Statistics

For all statistical analyses, confirm that the following items are present in the figure legend, table legend, main text, or Methods section.

n/a	Confirmed	
☐	☒	The exact sample size (*n*) for each experimental group/condition, given as a discrete number and unit of measurement
☐	☒	A statement on whether measurements were taken from distinct samples or whether the same sample was measured repeatedly
☐	☒	The statistical test(s) used AND whether they are one- or two-sided<br>*Only common tests should be described solely by name; describe more complex techniques in the Methods section.*
☐	☒	A description of all covariates tested
☐	☒	A description of any assumptions or corrections, such as tests of normality and adjustment for multiple comparisons
☐	☒	A full description of the statistical parameters including central tendency (e.g. means) or other basic estimates (e.g. regression coefficient) AND variation (e.g. standard deviation) or associated estimates of uncertainty (e.g. confidence intervals)
☐	☒	For null hypothesis testing, the test statistic (e.g. *F*, *t*, *r*) with confidence intervals, effect sizes, degrees of freedom and *P* value noted<br>*Give P values as exact values whenever suitable.*
☒	☐	For Bayesian analysis, information on the choice of priors and Markov chain Monte Carlo settings
☒	☐	For hierarchical and complex designs, identification of the appropriate level for tests and full reporting of outcomes
☐	☒	Estimates of effect sizes (e.g. Cohen's *d*, Pearson's *r*), indicating how they were calculated

Our web collection on statistics for biologists contains articles on many of the points above.

Software and code

Policy information about availability of computer code

Data collection	No data collection was performed, and hence no software was used for data collection.
Data analysis	The Microbiome Modeling Toolbox (COBRA toolbox commit: 71c117305231f77a0292856e292b95ab32040711) (https://github.com/opencobra/cobratoolbox). All metabolic modeling computations were performed in MATLAB version 2019a (Mathworks, Inc.), using the IBM CPLEX (IBM, Inc.) solver, version 12.10.0.<br>Python 3.7.9 was used for all statistical analysis using the following packages:<br>SHAP version 0.35.0<br>scipy version 1.5.2<br>umap version 0.4.6<br>skbio version 0.5.6<br>statsmodels version 0.12.1<br>R version 3.6.1<br>rpy2 version 3.4.2<br>lightgbm version 3.2.1<br>scikit-learn version 0.24.2<br>scikit-learn-extra version 0.2.0<br>pandas version 1.1.5<br>numpy version 1.18.5<br>usearch version 11.0.667<br>PICrust2 version 2.4.1

AMON version 1.0.0
Scripts to reproduce the analysis are available at the GitHub repository: https://github.com/korem-lab/PTB_Metabs_2021

For manuscripts utilizing custom algorithms or software that are central to the research but not yet described in published literature, software must be made available to editors and reviewers. We strongly encourage code deposition in a community repository (e.g. GitHub). See the Nature Portfolio guidelines for submitting code & software for further information.

Data

Policy information about availability of data

All manuscripts must include a data availability statement. This statement should provide the following information, where applicable:

- Accession codes, unique identifiers, or web links for publicly available datasets
- A description of any restrictions on data availability
- For clinical datasets or third party data, please ensure that the statement adheres to our policy

The 16S rRNA gene amplicon sequencing data and the associated samples and subjects' metadata analyzed in this study are publicly available in the database of Genotypes and Phenotypes (dbGaP) under accession number phs001739.v1.p1 as well as in Supplementary Data 2 of ref. 14. Raw metabolomics data is available in Table S1. Mass spectral data is available from MetaboLights under accession number MTBLS702 (https://www.ebi.ac.uk/metabolights/MTBLS702). Additional information regarding xenobiotics is provided in Table S13. The KEGG Database is available at https://www.genome.jp/kegg/ and the AGORA models are available at vmh.life.

Human research participants

Policy information about studies involving human research participants and Sex and Gender in Research.

Reporting on sex and gender: Analysis regarding sex is inapplicable as this study only included pregnant females. Additional information regarding gender is not available to us.

Population characteristics: This was a nested case-control study from the Motherhood & Microbiome cohort. Sample selection is detailed below. Population characteristics are provided in Table 1. In brief, the study included 80 women who delivered preterm and 152 women who delivered at term. Study groups were matched in all relevant characteristics, including age (29±6 vs. 28±6), BMI (30.1±7.8 vs. 30.6±7.2), and maternal race (71.25% Black women vs. 76.3% Black women.).

Recruitment: This study did not involve recruitment. We analyzed banked samples from the previously collected and described Motherhood & Microbiome (M&M) cohort (NCT02030106), described in Elovitz et al, Nat. Commun. 2019. The M&M cohort recruited 2,000 women with a singleton pregnancy prior to 20 weeks of gestation. Women were followed to delivery, and spontaneous preterm birth was defined as delivery before 37 weeks of gestation with a presentation of cervical dilation and/or premature rupture of membranes.

Ethics oversight: All participants provided written informed consent and the study was approved by the Institutional Review Board at the University of Pennsylvania (IRB #818914) and the University of Maryland School of Medicine (HP-00045398).

Note that full information on the approval of the study protocol must also be provided in the manuscript.

Field-specific reporting

Please select the one below that is the best fit for your research. If you are not sure, read the appropriate sections before making your selection.

☒ Life sciences ☐ Behavioural & social sciences ☐ Ecological, evolutionary & environmental sciences

For a reference copy of the document with all sections, see nature.com/documents/nr-reporting-summary-flat.pdf

Life sciences study design

All studies must disclose on these points even when the disclosure is negative.

Sample size: We analyzed banked samples from the previously collected and described Motherhood & Microbiome (M&M) cohort. For this study, out of all women with available microbiome data, all available samples from weeks 20-24 of gestation from women who delivered preterm spontaneously (N = 80) were selected, in addition to samples from 152 matched controls (1:~2) who delivered at term. Sample size was selected by availability and not by sample size calculation.

Data exclusions: No data was excluded.

Replication: The metabolomics-based prediction algorithm described in the manuscript was successfully validated in two external cohorts.
These two external cohorts were ill-suited to replicate specific associations: only a small fraction of the metabolites that were measured in our dataset were also measured in the validation cohorts (18% and 32%). Of the 11 significant associations in Figure 2a, 6 can be validated in the Ghartey 2015 cohort (which is mostly composed of White women), and 9 can be validated in the Ghartey 2017 cohort (which is mostly composed of Black women). Of these, only one and two metabolites were measured in the Ghartey 2015 and 2017 cohorts, respectively. Of these three associations that could be replicated, none were significant. We attribute this to differences in study design, inclusion criteria, and

target population, as well as to the small sample size of both of these cohorts.
The manuscript also details numerous findings that were supported by existing literature.

Randomization	The study was an observational study with no intervention and therefore allocation and randomization is not relevant to the study.
Blinding	No blinding was performed for outcome assessment as this was an observational study. Metabolon Inc. were blinded to the outcome assessment of each sample during metabolomics processing. Furthermore, clinical metadata was required for the multi-omic analyses performed.

Reporting for specific materials, systems and methods

We require information from authors about some types of materials, experimental systems and methods used in many studies. Here, indicate whether each material, system or method listed is relevant to your study. If you are not sure if a list item applies to your research, read the appropriate section before selecting a response.

Materials & experimental systems

n/a	Involved in the study	
☒	☐	Antibodies
☒	☐	Eukaryotic cell lines
☒	☐	Palaeontology and archaeology
☒	☐	Animals and other organisms
☒	☐	Clinical data
☒	☐	Dual use research of concern

Methods

n/a	Involved in the study	
☒	☐	ChIP-seq
☒	☐	Flow cytometry
☒	☐	MRI-based neuroimaging
